# Supplementary material for: The interactome of intact mitochondria by cross-linking mass spectrometry provides evidence for coexisting respiratory supercomplexes
Source: Mol Cell Proteomics. 2017 Dec 8;17(2):216–32. doi: 10.1074/mcp.RA117.000470 (PMC5795388; doi:10.1074/mcp.RA117.000470)
Supplement: Supplemental Data [file supp_RA117.000470_133922_0_supp_23978_fzffwf.zip › spectra_annotation/mito_DR_spectra_annotation/55-1-8-1-1-1.pdf]

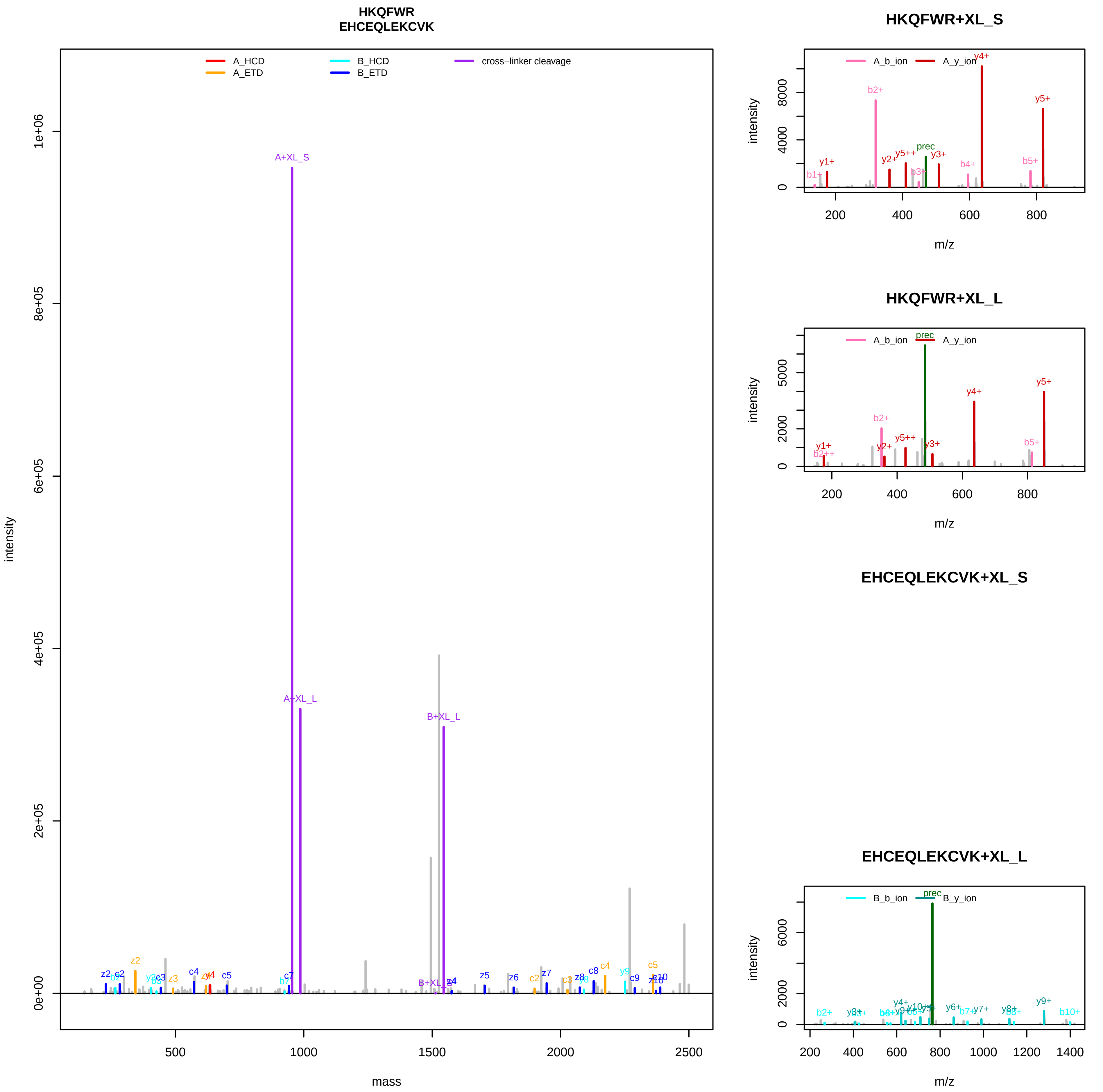
HKQFWR
EHCEQLEKCVK
A_HCD
A_ETD
B_HCD
B_ETD
cross−linker cleavage
intensity
mass
0e+00
2e+05
4e+05
6e+05
8e+05
1e+06
500
1000
1500
2000
2500
A+XL_S
A+XL_L
B+XL_L
B+XL_S
z2
c2
b2
y3
b3
c3
z3
c4
y4
c5
b7
c7
z4
c4
z5
z6
c2
z7
c3
z8
y8
c8
c4
y9
c9
c5
z10
b10
HKQFWR+XL_S
A_b_ion
A_y_ion
intensity
m/z
0
4000
8000
200
400
600
800
b1+
y1+
b2+
y2+
y5++
b3+
prec
y3+
b4+
y4+
b5+
y5+
HKQFWR+XL_L
A_b_ion
A_y_ion
intensity
m/z
0
2000
5000
200
400
600
800
y1+
b2++
b2+
y2+
y5++
prec
y3+
y4+
b5+
y5+
EHCEQLEKCVK+XL_S
EHCEQLEKCVK+XL_L
B_b_ion
B_y_ion
intensity
m/z
0
2000
6000
200
400
600
800
1000
1200
1400
b2+
y3+
b3+
b4+
y4+
y9+
y10+
b5+
y5+
prec
y6+
b7+
y7+
y8+
b8+
y9+
b10+
